# Dataset of de novo assembly and functional annotation of the transcriptomes of three native oleaginous microalgae from the Peruvian Amazon

**DOI:** 10.1016/j.dib.2020.105917

**Published:** 2020-06-21

**Authors:** Marianela Cobos, Hicler N. Rodríguez, Segundo L. Estela, Carlos G. Castro, J. Dylan Maddox, Jae D. Paredes, Juan R. Saldaña, Álvaro B. Tresierra, Juan C. Castro

**Affiliations:** a Laboratorio de Biotecnología y Bioenergética (LBB), Universidad Científica del Perú (UCP), Av. Abelardo Quiñones Km 2.5, San Juan Bautista, Loreto, 16006, Perú; b Unidad Especializada de Biotecnología, Centro de Investigaciones de Recursos Naturales de la Amazonía (CIRNA), Universidad Nacional de la Amazonia Peruana (UNAP), Psje. Los Paujiles S/N, San Juan Bautista, Loreto, 16000, Perú; c Pritzker Laboratory for Molecular Systematics and Evolution, Field Museum of Natural History, 1400 S. Lake Shore Drive, Chicago, IL 60605, USA; d Environmental Sciences, American Public University System, Charles Town, WV 25414, USA; e Departamento Académico de Ciencias Biomédicas y Biotecnología, Facultad de Ciencias Biológicas, Universidad Nacional de la Amazonia Peruana (UNAP), Ciudad Universitaria - Zungarococha, San Juan Bautista, Loreto, 16000, Perú

**Keywords:** Bioactive compounds, Biofuel, Gene expression profiling, Metabolic pathways, Microalgae, RNA-seq

## Abstract

Microalgae are photosynthetic organisms with cosmopolitan distribution (i.e., marine, freshwater and terrestrial habitats) and possess a great diversity of species [1] and consequently an immense variation in biochemical compositions [2]. To date genomic information is available mainly from the model green microalga *Chlamydomonas reinhardtii*[3]. Here we provide the dataset of a de novo assembly and functional annotation of the transcriptomes of three native oleaginous microalgae from the Peruvian Amazon. Native oleaginous microalgae species *Ankistrodesmus* sp., *Chlorella* sp., and *Scenedesmus* sp. were cultured in triplicate using Chu-10 medium with or without a source of nitrate (NaNO_3_). Total RNA was purified, the cDNA libraries were constructed and sequenced as paired-end reads on an Illumina HiSeq™2500 platform. Transcriptomes were de novo assembled using Trinity v2.9.1. A total of 48,554 transcripts (range from 250 to 7966 bp; N50 = 1047) for *Ankistrodesmus* sp., 108,126 transcripts (range from 250 to 8160 bp; N50 = 1090) for *Chlorella* sp., and 77,689 transcripts (range from 250 to 8481 bp; N50 = 1281) for *Scenedesmus* sp. were de novo assembled. Completeness of the assembled transcriptomes were evaluated with the Benchmarking Universal Single-Copy Orthologs (BUSCO) software v2/v3. Functional annotation of the assembled transcriptomes was conducted with TransDecoder v3.0.1 and the web-based platforms Kyoto Encyclopedia of Genes and Genomes (KEGG) Automatic Annotation Server (KAAS) and FunctionAnnotator. The raw reads were deposited into NCBI and are accessible via BioProject accession number PRJNA628966 (https://www.ncbi.nlm.nih.gov/bioproject/PRJNA628966) and Sequence Read Archive (SRA) with accession numbers: SRX8295665 (https://www.ncbi.nlm.nih.gov/sra/SRX8295665), SRX8295666 (https://www.ncbi.nlm.nih.gov/sra/SRX8295666), SRX8295667 (https://www.ncbi.nlm.nih.gov/sra/SRX8295667), SRX8295668 (https://www.ncbi.nlm.nih.gov/sra/SRX8295668), SRX8295669 (https://www.ncbi.nlm.nih.gov/sra/SRX8295669), and SRX8295670 (https://www.ncbi.nlm.nih.gov/sra/SRX8295670). Additionally, transcriptome shotgun assembly sequences and functional annotations are available via Discover Mendeley Data (https://data.mendeley.com/datasets/47wdjmw9xr/1).

Specifications table

**Table:** 

Subject	Genetics, Genomics and Molecular Biology
Specific subject area	Transcriptomics
Type of data	Figures, Table, raw paired-end sequencing data, transcriptome shotgun assembly sequence database, and functional annotation results.
How data were acquired	Total RNA was purified from three native oleaginous microalgae species (*Ankistrodesmus* sp., *Chlorella* sp., and *Scenedesmus* sp.) that were previously cultured in triplicate using Chu-10 medium with or without a source of nitrate. Libraries were constructed using standardized protocols and paired-end sequenced on an Illumina HiSeq™2500 platform.
Data format	Raw data files in fastq.gz format were deposited into NCBI database and are available at BioProject accession number PRJNA628966 (https://www.ncbi.nlm.nih.gov/bioproject/PRJNA628966) and SRA accession numbers:
	SRX8295665 (https://www.ncbi.nlm.nih.gov/sra/SRX8295665)
	SRX8295666 (https://www.ncbi.nlm.nih.gov/sra/SRX8295666)
	SRX8295667 (https://www.ncbi.nlm.nih.gov/sra/SRX8295667)
	SRX8295668 (https://www.ncbi.nlm.nih.gov/sra/SRX8295668)
	SRX8295669 (https://www.ncbi.nlm.nih.gov/sra/SRX8295669)
	SRX8295670 (https://www.ncbi.nlm.nih.gov/sra/SRX8295670)
	Also, transcriptome shotgun assembly sequences database (fasta.gz format) and functional annotation results are openly accessible at Discover Mendeley Data (https://data.mendeley.com/datasets/47wdjmw9xr/1).
Parameters for data collection	Total RNA was purified from three native oleaginous microalgae species (*Ankistrodesmus* sp., *Chlorella* sp., and *Scenedesmus* sp.) that were previously cultured in triplicate using Chu-10 medium with or without a source of nitrate. Libraries were constructed using standardized protocols and paired-end sequenced on an Illumina HiSeq™2500 platform.
Description of data collection	High quality fastq reads were de novo assembled with Trinity v2.9.1. Completeness of the assembled transcriptomes were tested with the Benchmarking Universal Single-Copy Orthologs (BUSCO) software v2/v3 as implemented in the web-based server gVolante (https://gvolante.riken.jp/). Further, assembled transcripts were functionally annotated with TransDecoder v3.0.1, Kyoto Encyclopedia of Genes and Genomes (KEGG) Automatic Annotation Server (KAAS) v2.1 (https://www.genome.jp/tools/kaas/) and FunctionAnnotator (http://fa.cgu.edu.tw/index.php).
Data source location	Institution: Universidad Científica del Perú (UCP)
	City/Town/Region: Iquitos/Maynas/Loreto Region
	Country: Peru
	Latitude and longitude (and GPS coordinates) for collected samples/data:
	The three native oleaginous microalgae were obtained from the microalgae culture collection of the UCP, which were previously isolated from the Amazon river (03°41′0.60" S, 73°14′8.88" W), the Itaya river (03°43′1.56" S, 73°14′17.88" W), and the Nanay river (3°42′0.36" S, 73°15′32.04" W).
Data accessibility	Raw data files in fastq.gz format are available from NCBI under BioProject accession number PRJNA628966 (https://www.ncbi.nlm.nih.gov/bioproject/PRJNA628966) and SRA accession numbers:
	SRX8295665 (https://www.ncbi.nlm.nih.gov/sra/SRX8295665)
	SRX8295666 (https://www.ncbi.nlm.nih.gov/sra/SRX8295666)
	SRX8295667 (https://www.ncbi.nlm.nih.gov/sra/SRX8295667)
	SRX8295668 (https://www.ncbi.nlm.nih.gov/sra/SRX8295668)
	SRX8295669 (https://www.ncbi.nlm.nih.gov/sra/SRX8295669)
	SRX8295670 (https://www.ncbi.nlm.nih.gov/sra/SRX8295670)
	Also, transcriptome shotgun assembly sequences database (fasta.gz format) and functional annotation results are hosted in the public repository Discover Mendeley Data (https://data.mendeley.com/datasets/47wdjmw9xr/1).

## Value of the data

• These are the first datasets of the de novo assembly and functional annotation of the transcriptomes of three native oleaginous microalgae from the Peruvian Amazon.
• These data provide valuable information on identified genes encoding enzymes involved in the biosynthesis of lipids and carbohydrates appropriates for biofuel production by the native oleaginous microalgae from the Peruvian Amazon.
• The transcriptome datasets can be used to elucidate anabolic pathways involved in the production of human essential nutrients and the great diversity of health-promoting compounds by the native oleaginous microalgae from the Peruvian Amazon.

## 1 Data description

**Fig. 1:**
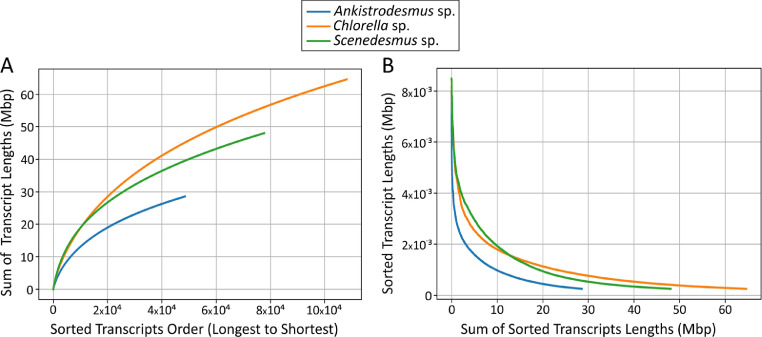
Cumulative length (A) and sorted transcripts length (B) of the de novo assembled transcriptomes of the native oleaginous microalgae *Ankistrodesmus* sp., *Chlorella* sp., and *Scenedesmus* sp.

**Fig. 2:**
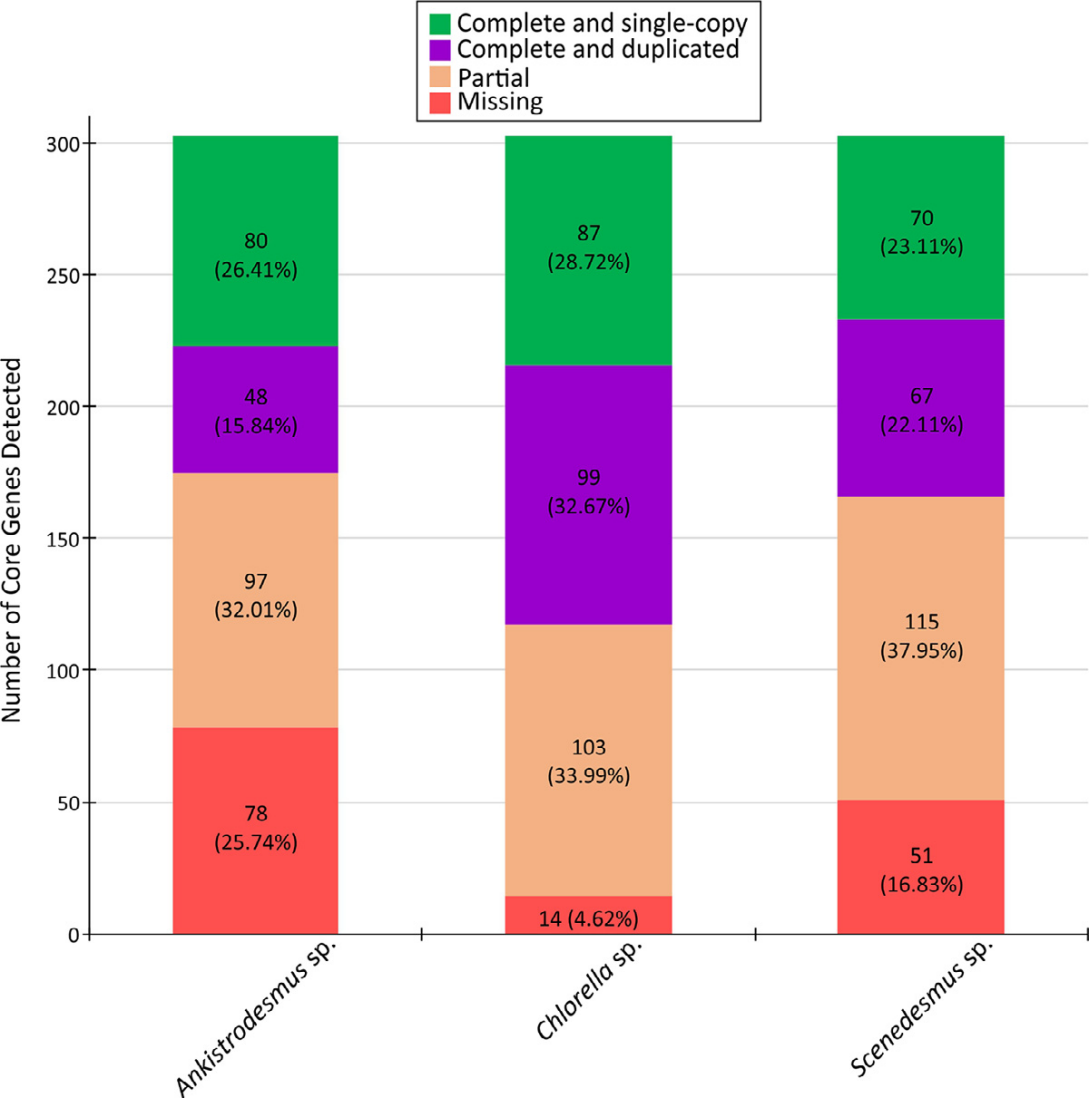
Completeness scores of the de novo assembled transcriptomes of the native oleaginous microalgae *Ankistrodesmus* sp., *Chlorella* sp., and *Scenedesmus* sp.

Included in this dataset are the de novo assembly and functional annotation of the transcriptomes of the native oleaginous microalgae *Ankistrodesmus* sp., *Chlorella* sp., and *Scenedesmus* sp. that were previously cultured in triplicate using Chu-10 medium with or without a source of nitrate (NaNO_3_). Total RNA was purified from each microalgae species in both cultured conditions, then pooled in equimolar ratios to construct the six cDNA libraries and paired-end sequenced on an Illumina HiSeq™2500 platform. De novo transcriptome assembly was conducted using Trinity v2.9.1 (Fig. S1). A total of 48,554 transcripts (range from 250 to 7966 bp; N50 = 1047) for *Ankistrodesmus* sp., 108,126 transcripts (range from 250 to 8160 bp; N50 = 1090) for *Chlorella* sp., and 77,689 transcripts (range from 250 to 8481 bp; N50 = 1281) for *Scenedesmus* sp. were de novo assembled (Fig. 1, Table 1). Completeness of the assembled transcriptomes were tested with the Benchmarking Universal Single-Copy Orthologs (BUSCO) software v2/v3, which revealed that of the 303 Eukaryote core genes queried, from 225 to 289 were detected (complete + partial from 74.26 to 95.38%) and from 4.62 to 25.74% were missing (Fig. 2) with an average number of orthologs per core genes of 1.49, 1.77 and 1.67 for *Ankistrodesmus* sp., *Chlorella* sp., and *Scenedesmus* sp., respectively.

**Table 1:** Assembly parameters of the de novo assembled transcriptomes of the native oleaginous microalgae *Ankistrodesmus* sp., *Chlorella* sp., and *Scenedesmus* sp.

Assembly Parameter	Microalgae Strain		
	*Ankistrodesmus* sp.	*Chlorella* sp.	*Scenedesmus* sp.
# contigs (≥ 250 bp)	48,554	108,126	77,689
# contigs (≥ 500 bp)	18,672	40,957	27,811
# contigs (≥ 1000 bp)	6003	13,701	10,088
Total length (≥ 250 bp)	28,571,945	64,599,906	48,044,789
Total length (≥ 1000 bp)	9675,975	22,903,831	19,061,876
Largest contig	7966	8160	8481
Total length	18,360,467	41,615,261	31,088,244
GC (%)	64.28	55.64	54.45
N50	1047	1090	1281
N75	709	726	759
L50	5519	11,691	6965
L75	10,895	23,518	15,010
# N's per 100 kbp	0.0	0.0	0.0

**Fig. 3:**
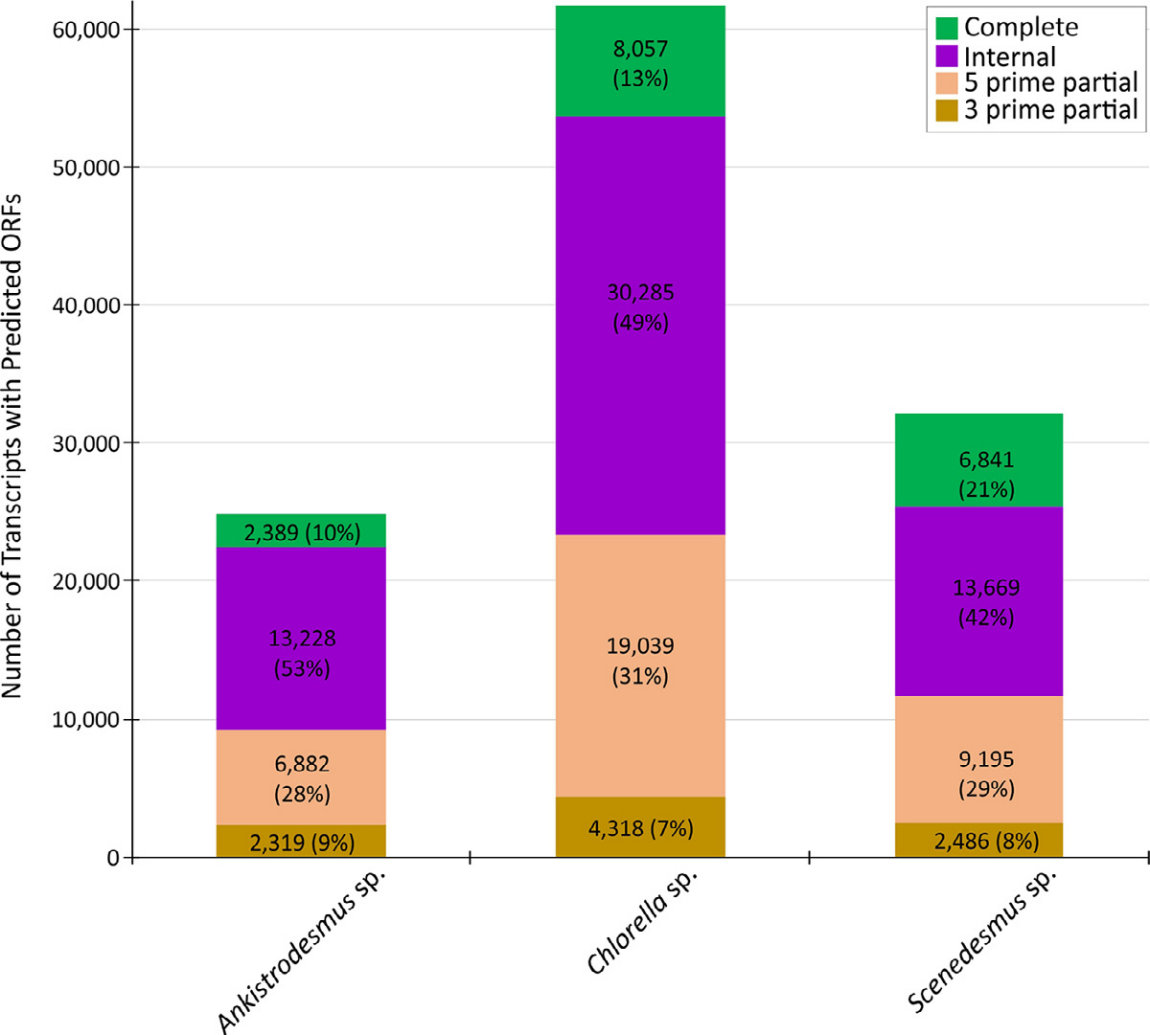
Summary of ORFs predicted in the de novo assembled transcriptomes of the native oleaginous microalgae *Ankistrodesmus* sp., *Chlorella* sp., and *Scenedesmus* sp.

The de novo assembled transcriptomes were functionally annotated with TransDecoder v3.0.1 and the web-based platforms Kyoto Encyclopedia of Genes and Genomes (KEGG) Automatic Annotation Server (KAAS) and FunctionAnnotator. TransDecoder predicted the four open reading frames categories (Fig. 3) as complete (from 10 to 21%), internal (from 42 to 53%), 5 prime partial (from 28 to 31%), and 3 prime partial (from 7 to 9%). Also, from 17,138 to 39,868 assembled transcripts were assigned with gene ontology terms (Table S1). Kyoto Encyclopedia of Genes and Genomes (KEGG) Automatic Annotation Server (KAAS) assigned KEGG Orthology (KO) IDs to 11,347 transcripts (2626 unique KO) for *Ankistrodesmus* sp., to 25,128 transcripts (3334 unique KO) for *Chlorella* sp., and to 17,627 transcripts (2942 unique KO) for *Scenedesmus* sp. BRITE hierarchies (KEGG modules, KEGG orthology, and KEGG reaction modules) and 135 metabolic pathway maps were generated with 2501, 2929, and 2702 enzymes/proteins mapped for *Ankistrodesmus* sp., *Chlorella* sp., and *Scenedesmus* sp., respectively (Table S1). Finally, from 30,412 to 62,012 best hits against the NCBI non-redundant protein database were obtained, from 917 to 2205 enzymes were identified, and from 13,337 to 30,633 transcripts encoding at least one domain region in proteins were identified (Table S2).

Raw data files in fastq.gz format are available from NCBI under BioProject accession number PRJNA628966 (https://www.ncbi.nlm.nih.gov/bioproject/PRJNA628966) and SRA accession numbers:

SRX8295665 (https://www.ncbi.nlm.nih.gov/sra/SRX8295665), SRX8295666 (https://www.ncbi.nlm.nih.gov/sra/SRX8295666), SRX8295667 (https://www.ncbi.nlm.nih.gov/sra/SRX8295667), SRX8295668 (https://www.ncbi.nlm.nih.gov/sra/SRX8295668), SRX8295669 (https://www.ncbi.nlm.nih.gov/sra/SRX8295669), and SRX8295670 (https://www.ncbi.nlm.nih.gov/sra/SRX8295670). Additionally, transcriptome shotgun assembly sequences and functional annotations are available via Discover Mendeley Data (https://data.mendeley.com/datasets/47wdjmw9xr/1).

## 2 Experimental design, materials, and methods

### 2.1 Microalgae culture and harvest

Three native oleaginous microalgae *Ankistrodesmus* sp. UCP001 (isolated from the Itaya river [03°43′1.56″ S, 73°14′17.88″ W]), *Chlorella* sp. UCP002 (isolated from the Amazon river [03°41′0.60″ S, 73°14′8.88″ W]) and *Scenedesmus* sp. UCP003 (isolated from the Nanay river [3°42′0.36″ S, 73°15′32.04″ W]) were obtained from the Microalgae Culture Collection of the Universidad Científica del Perú (UCP). Microalgae cells were inoculated (OD_680_ 0.10) in 100 mL of Chu-10 medium in a 250 mL Erlenmeyer flask and cultured for two weeks in a controlled culture room under the following conditions: temperature at 25 ± 1 °C, 12/12 h light/dark photoperiod, light intensity at 100 µmol photons m^−2^ s^−1^ supplied by cool-white fluorescent lamps, and continuous agitation at 150 rpm. Cultures were harvested by centrifugation (2000 × g, 10 min, 4 °C) and the microalgae cells were rinsed two times with 45 mL of sterilized ultrapure water, centrifuged again and the supernatants were discarded. Further, microalgae cells were resuspended in saline solution (NaCl 0.9%) and inoculated (OD_680_ 0.2) in triplicate in 25 mL Chu-10 medium with or without a source of nitrate (NaNO_3_) in a 125 mL Erlenmeyer flask and cultured for one week as previously described. Further, microalgae cultures were harvested by centrifugation (2000 × *g*, 10 min, 4 °C) and the microalgae biomass was rinsed as previously described. Microalgae cells were immediately stored at −80 °C until further use. A flow diagram of methodological approaches is provided in the Supplementary material (Fig. S1).

### 2.2 Total RNA isolation, library preparation and next-generation DNA sequencing

Total RNA was isolated following the manufacturer's instructions using the RNeasy Plant Mini Kit (Qiagen, Hilden, Germany). Quantity and quality values of the total RNA were evaluated by spectrophotometric analysis using a Nanodrop 2000 Spectrophotometer and RNA integrity using a 2100 Bioanalyzer (Agilent, CA, USA). Total RNA obtained from each microalgae species and every culture condition were pooled in equimolar ratios to construct the six cDNA libraries. The cDNA libraries were constructed following the manufacturer's instructions of the TruSeq Stranded mRNA Sample Preparation Kit (Illumina, San Diego, USA), quantified with the Qubit™ dsDNA HS Assay Kit (Thermo Fisher Scientific, Waltham, USA) and paired-end sequenced (2 × 150 bp) on an Illumina HiSeq™2500 platform.

### 2.3 De novo assembly and functional annotation

Raw paired-end sequences were uploaded as FASTQ files to the Galaxy (https://usegalaxy.org/) bioinformatic platform. In this bioinformatic platform the quality of the fastq files were evaluated with FastQC [4] and the adaptor sequences, low quality bases (≤ Q20) and short sequences (≤ 50 bp in length) were trimmed with Trimommatic (Galaxy version 0.38.0) [5]. High quality sequence reads were de novo assembled using Trinity (Galaxy version 2.9.1) [6] with default parameters and a minimum contig length of 250 bp. Completeness of assembled transcripts was evaluated using the Benchmarking Universal Single-Copy Orthologs (BUSCO) software v2/v3 [7] as implemented in the web-based server gVolante [8].

Functional annotations of the de novo assembled transcriptomes were conducted with the following bioinformatic tools: 1) TransDecoder (Galaxy version 3.0.1) [9] to predict Open Reading Frames (ORFs) and to obtain protein sequences of at least 100 amino acids in length; 2) Kyoto Encyclopedia of Genes and Genomes (KEGG) Automatic Annotation Server (KAAS) v2.1 (https://www.genome.jp/tools/kaas/) with default threshold bit-score value of 60, single-directional best hit (SBH) method, BLASTx program, and genes dataset of green algae (*Auxenochlorella protothecoides, Chlamydomonas reinhardtii, Micromonas commoda, Micromonas pusilla, Monoraphidium neglectum, Ostreococcus lucimarinus*, and *Ostreococcus tauri*), red algae (*Cyanidioschyzon merolae, Chondrus crispus*, and *Galdieria sulphuraria*), and the eudicot *Arabidopsis thaliana* to assign KEGG Orthology (KO) IDs, to obtain BRITE hierarchies, and to generate the metabolic pathway maps; and 3) FunctionAnnotator (http://fa.cgu.edu.tw/index.php) to obtain the best hit in NCBI non-redundant protein database (Taxonomic distribution and GO function annotation [Blast2GO]), to predict enzymes (PRIAM database), and to identify domain regions in proteins (Domain finder).

## Declaration of Competing Interest

The authors declare that they have no known competing financial interests or personal relationships which have, or could be perceived to have, influenced the work reported in this article.

## Appendix Supplementary materials

Image, image 1

Image, application 2

Image, application 3

Image, application 4
