# Supplementary figures and images for: Dataset of de novo assembly and functional annotation of the transcriptomes of three native oleaginous microalgae from the Peruvian Amazon
Source: Data Brief. 2020 Jun 21;31:105917. doi: 10.1016/j.dib.2020.105917 (PMC7327806; doi:10.1016/j.dib.2020.105917)

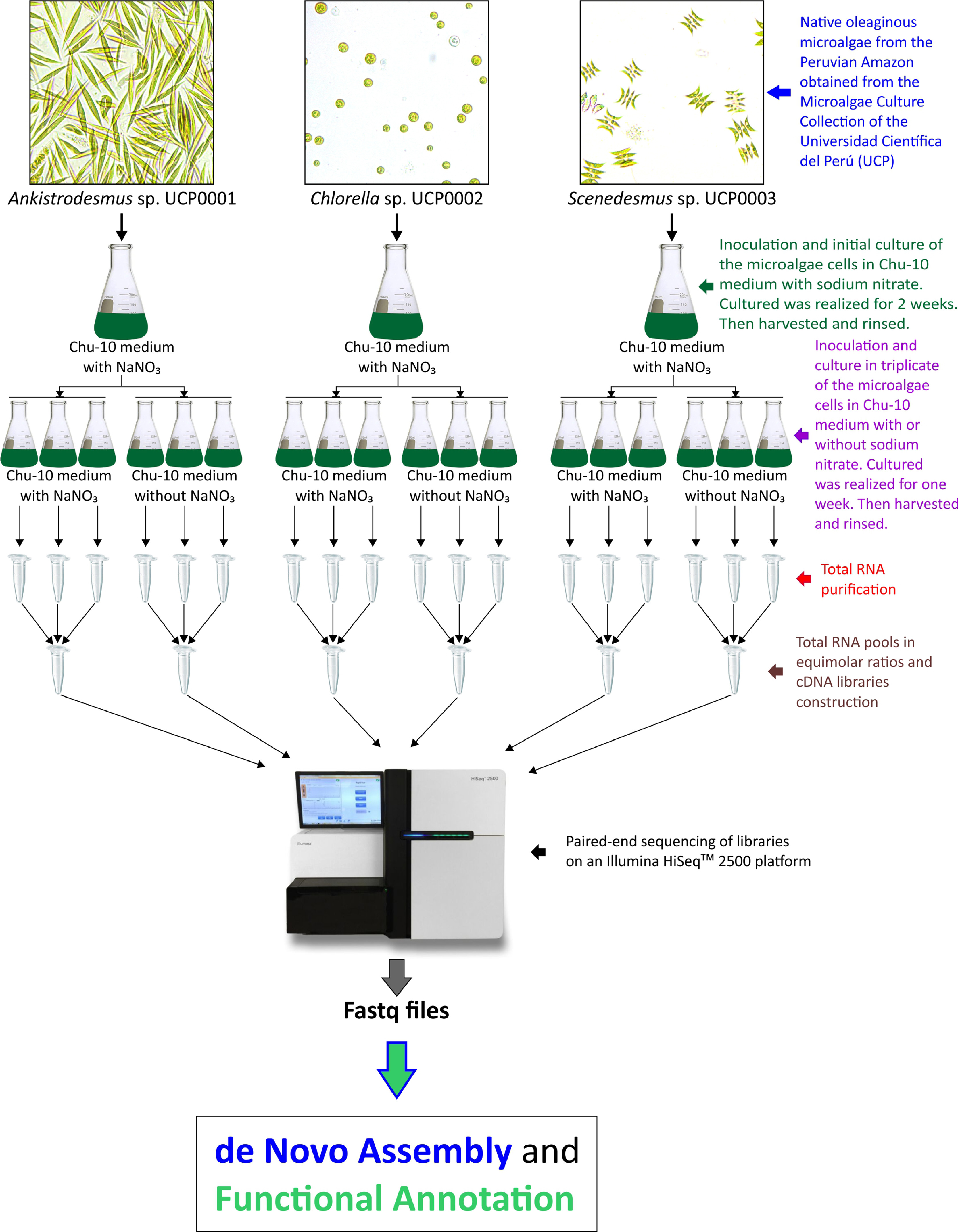

Supplement: Supplementary file 1 [file mmc1.jpg]
